# Breast Cancer Stigma Scale: A Reliable and Valid Stigma Measure for Patients With Breast Cancer

**DOI:** 10.3389/fpsyg.2022.841280

**Published:** 2022-06-10

**Authors:** Xiaofan Bu, Shuangshuang Li, Andy S. K. Cheng, Peter H. F. Ng, Xianghua Xu, Yimin Xia, Xiangyu Liu

**Affiliations:** ^1^Nursing Teaching and Research Section, Hunan Cancer Hospital, The Affiliated Cancer Hospital of Xiangya School of Medicine, Central South University, Changsha, China; ^2^Department of Nursing, The Second Xiangya Hospital of Central South University, Changsha, China; ^3^Department of Rehabilitation Sciences, The Hong Kong Polytechnic University, Hong Kong, Hong Kong SAR, China; ^4^Department of Computing, The Hong Kong Polytechnic University, Hong Kong, Hong Kong SAR, China; ^5^Department of Health Service Center, Hunan Cancer Hospital, The Affiliated Cancer Hospital of Xiangya School of Medicine, Central South University, Changsha, China

**Keywords:** breast cancer, stigma, scale, development, validation, reliability

## Abstract

**Purpose:**

This study aims to develop and validate a stigma scale for Chinese patients with breast cancer.

**Methods:**

Patients admitted to the Affiliated Cancer Hospital of Xiangya School of Medicine, Central South University, for breast cancer treatment participated in this study. Development of the Breast Cancer Stigma Scale involved the following procedures: literature review, interview, and applying a theoretical model to generate items; the Breast Cancer Stigma Scale’s content validity was assessed by a Delphi study (*n* = 15) and feedback from patients with breast cancer (*n* = 10); exploratory factor analysis (*n* = 200) was used to assess the construct validity; convergent validity was assessed with the Social Impact Scale (*n* = 50); internal consistency Cronbach’s α (*n* = 200), split-half reliability (*n* = 200), and test–retest reliability (*N* = 50) were used to identify the reliability of the scale.

**Results:**

The final version of the Breast Cancer Stigma Scale consisted of 15 items and showed positive correlations with the Social Impact Scale (ρ = 0.641, *P* < 0.001). Exploratory factor analysis (EFA) revealed four components of the Breast Cancer Stigma Scale: self-image impairment, social isolation, discrimination, and internalized stigma, which were strongly related to our perceived breast cancer stigma model and accounted for 69.443% of the total variance. Cronbach’s α for the total scale was 0.86, and each subscale was 0.75–0.882. The test–retest reliability with intra-class correlation coefficients of the total scale was 0.947 (*P* < 0.001), and split-half reliability with intra-class correlation coefficients of the total scale was 0.911 (*P* < 0.001). The content validity index (CVI) was 0.73–1.0.

**Conclusion:**

The newly developed Breast Cancer Stigma Scale offers a valid and reliable instrument for assessing the perceived stigma of patients with breast cancer in clinical and research settings. It may be helpful for stigma prevention in China.

## Introduction

Breast cancer is currently the most common cancer type experienced by women worldwide, with an estimated 2.3 million new cases in 2020 (Sung et al., 2021). Approximately 11% of all breast cancer cases worldwide occur in China, and the incidence has increased rapidly in recent decades (Li et al., 2016). The 5-year relative survival rate for individuals with breast cancer is approximately 82% (Zeng et al., 2018). Despite the improved prognosis, adverse effects (e.g., loss of breasts, visible scarring, hair loss, and lymphedema) from surgery, chemotherapy, and radiotherapy can be significantly disfiguring and can negatively affect patients’ lives (Suwankhong and Liamputtong, 2016). In addition, psychological stressors from adverse effects cause them to experience stigma and limit their social interactions due to changes in body image and others’ perception of them as “abnormal” (Rajasooriyar et al., 2021).

Stigma is typically a social process, experienced or anticipated, and is characterized by exclusion, rejection, blame, or devaluation that results from experience, perception, or reasonable anticipation of an adverse social judgment regarding a person or group (Lebel and Devins, 2008). The prevalence rate of perceived cancer-related stigma ranges from 5 to 90% (Ohaeri et al., 1998; Cho et al., 2013; Phelan et al., 2013; Fujisawa et al., 2020). Approximately 76.7 and 8.7% of the breast cancer survivors report moderate and high stigma levels, respectively (Jin et al., 2021). Perceptions related to one’s own body may impact the sense of identity, self-esteem, acceptance, sexuality, and perceived stigma of women (Tripathi et al., 2017). The main factors influencing stigma in China were personal acceptance of the disease and body image (Jin et al., 2021). Physical appearance impacts self-esteem, depression, and a tendency toward social isolation.

Breasts are considered a symbol of physical and sexual attractiveness and femininity. The psychological ramifications of a mastectomy can be substantial for women. They face distress and disfigurement due to missing or asymmetric breasts (Fang et al., 2013). Moreira and Canavarro (2010) reported that those treated with mastectomy were more dissatisfied and felt more ashamed of their appearance than those who had undergone breast-conserving surgery. Breast reconstruction offers an alternative opportunity for those who require mastectomy and improves women’s wellbeing and quality of life (Fang et al., 2013). A meta-analysis also indicated that women undergoing mastectomy alone perceived higher levels of distress than those undergoing mastectomy with immediate reconstruction or delayed reconstruction (Fang et al., 2013). Permanent changes (e.g., scars or loss of breasts) in a woman’s body resulting from breast surgery contribute to the perception of stigma.

Chemotherapy-induced alopecia (CIA) is a distressing side effect for those undergoing adjuvant chemotherapy. Chemotherapy does improve the survival rate of the cancer population; however, severe adverse effects of chemotherapy limit the dose and treatment continuation. Certain classes of chemotherapy agents (e.g., alkylating agents, anthracyclines, antibiotics, antimetabolites, vinca alkaloids, and taxanes) for breast cancer are known to cause alopecia more readily (Chon et al., 2012). CIA causes physical and psychological distress to patients and attracts unwanted attention, significantly affecting self-esteem and social interactions. For some women, losing hair was found to be even more distressing than losing their breasts (Trusson and Pilnick, 2017). The inability to conceal a negative body image is possible for this disparity. While they can wear prosthetic breasts or particular clothes to shape their body image, breast is an integral part of physical appearance and symbolizes health, femininity, and attractiveness, and influences body image and identity.

A series of existing measures could be used to assess breast cancer-related stigma. The Perceived Devaluation-Discrimination Scale was developed for individuals with mental illness to measure the subjective feelings of failure and the feeling of being less intelligent than others or for individuals whose opinions need not be taken seriously (Link et al., 1991, 2001). The Social Impact Scale, a 24-item instrument developed by Fife in 2000 (Fife and Wright, 2000), was used to evaluate the feelings of stigma in persons with HIV/AIDS and cancer. The Internalized Stigma of Mental Illness Scale, developed by Ritsher et al. (2003) in collaboration with people with mental illnesses in 2003, was used to measure the subjective experience of stigma, with subscales measuring alienation, stereotype endorsement, perceived discrimination, social withdrawal, and stigma resistance. The Consumer Experiences of Stigma Questionnaire was developed by Wahl (1999) to measure the stigma of patients with mental illness. The translation and measurement properties of these four scales have provided a framework to create a scale for measuring the stigma of patients with breast cancer in China. However, their items may be too broad to reflect the stigma attached to a single illness. As breast cancer survivors have much higher rates of anxiety, depression, cognition impairment, and adverse effects, it is necessary to explore in-depth stigma among patients with breast cancer and develop a tool that contains items specifically associated with the assessment of breast cancer-related impairments. Undoubtedly, universal measurements lack sensitivity to patients who are coping with breast cancer, who undergo a major disruption in their life course that leads to changes in their concept of self. In order to understand the stigma status of these patients and try to develop interventions to help these stigmatized individuals, the initial step is to create an effective evaluation measurement specifically for women with breast cancer. Therefore, this study aimed to develop and validate a measurement tool that is sensitive to the stigma experienced by women with breast cancer.

## Materials and Methods

### Part 1: Conceptualization and Development of an Initial Item Pool

We referred to a published method of examining the construct validity of newly developed instruments for creating objective measurements (Clark and Watson, 2019). We constructed a model of the perceived stigma associated with breast cancer based on the conceptual model of perceived lung cancer-related stigma developed by Cataldo et al. (2011), as well as conducted a literature search and qualitative interviews. We extracted the question items, conducted two rounds of Delphi study, pilot testing, and made corrections. Thus, the main themes and item pool of the stigma scale for patients with breast cancer were generated through a multi-step process, described in the following sections.

#### Literature Search

Conducting a comprehensive literature review enables a clear articulation of how the proposed scale will either be a theoretical or an empirical improvement over existing measures or will fill a vital measurement gap (Clark and Watson, 2019). Therefore, we performed a thorough literature review to understand the dimensions of self-stigma unique to patients with breast cancer. PubMed, Web of Science, Embase, CINAHL for full text, CNKI, Wanfang, VIP, and CBM were systematically searched from each database’s inception to March 2020 to explore dimensions of self-stigma in patients with breast cancer. The final search strategy combined the terms breast cancer or its related words and also stigma or its related words.

#### Applying a Theoretical Model

**FIGURE 1:**
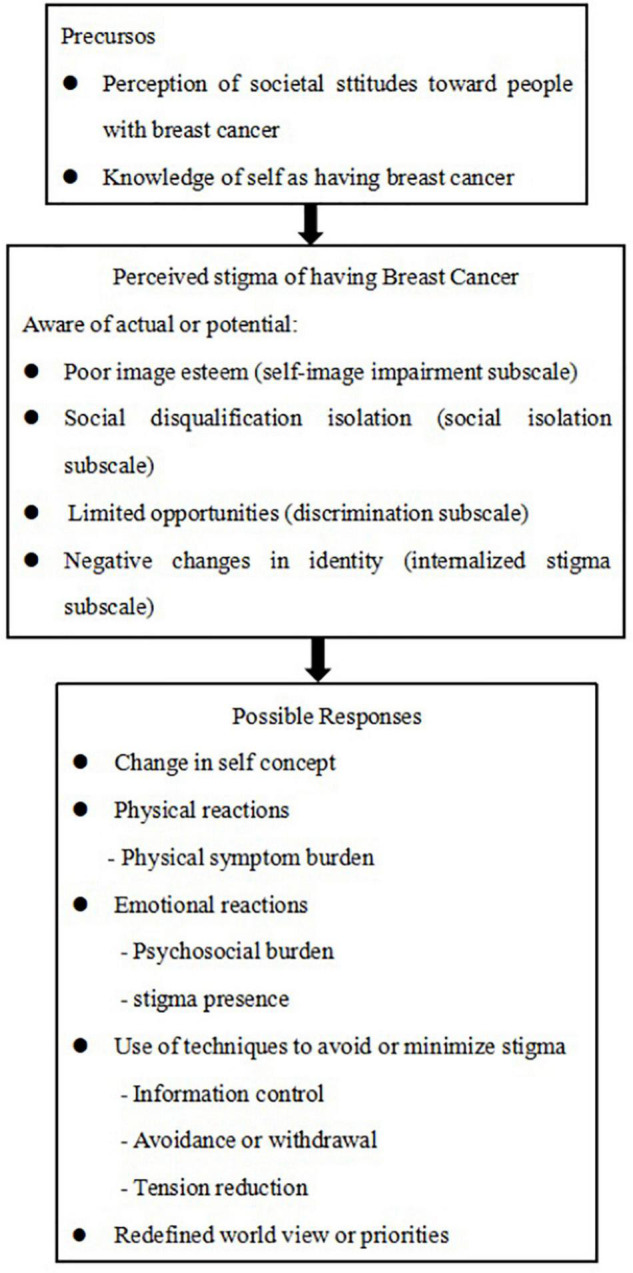
A model of perceived stigma in patients with breast cancer.

Some studies have researched perceived stigma among patients with cancer. However, early research only focused on discovering the self-stigma in their families. We developed a model of the perceived stigma associated with breast cancer based on the conceptual model of perceived lung cancer-related stigma developed by Cataldo et al. (2011). Figure 1 guided the development of items in the Breast Cancer Stigma Scale and described the process of perceived stigma among patients with breast cancer.

#### Qualitative Interviews to Explore Sub-Dimensions

The participant sample size depended on any beneficial information gleaned from the semi-structured interviews. Data collection ended once no further information was extracted. We conducted semi-structured interviews with 14 patients with breast cancer whose age, marital status, educational level, residence, stage of cancer, and surgery type varied to explore the appropriate sub-dimensions. The second author performed the interviews, which lasted 15–30 min per participant. Furthermore, the interviews were conducted face-to-face in a private and quiet room. All interviews were recorded by a digital voice recorder and subsequently transcribed verbatim within 24 h. The interviews were analyzed deductively, applying Colaizzi’s method of phenomenological analysis (Sanders, 2003). Four structural concepts were extracted related to the stigma process revealed through these interviews.

Self-image impairment, social isolation, discrimination, and internalized stigma were considered constructs of the stigma of patients with breast cancer.

#### Validating the Scale and Pilot Testing

We examined the initial items while using the Delphi method to explore the content validity of the Breast Cancer Stigma Scale. The Delphi method is an approach used to gain the most reliable consensus among a panel of experts by using a range of questionnaires (Keeney et al., 2001; Powell, 2003). We listed dimensions and the item pool during each Delphi round. Between each round, we revised the content based on the experts’ feedback. An invitation to participate in the consultation and a content description was sent by WeChat, email, or through in-person meetings to identified experts. Inclusion criteria for experts were those (1) with more than 10 years of working experience related to breast cancer nursing or psychology, (2) those with at least a professional title of associate professor, intermediate title, or above, and (3) experts who mastered in the psychometric assessment of a scale and who were willing to offer advice. Finally, 15 experts from nine provinces in China participated in the consultation. In total, eleven of these experts were in clinical nursing and nursing management in tertiary A general hospitals, two were psychologists, and two were nursing education experts in colleges. They reviewed the content and evaluated each item’s score independently using a 5-point Likert-type scale (ranging from “not important” to “very important”). A pilot test was performed on 10 participants by completing the 24-item questionnaire of scale items and it was with good validation.

### Phase II: Scale Validation

#### Participants

Patients who received treatment in the breast internal medicine department or breast surgery department in the Hunan Cancer Hospital were conveniently and consecutively recruited from September 2020 to February 2021. The ethical committees of the Hunan Cancer Hospital, approved this study. All the participants provided their informed consent. The inclusion criteria were those (1) ⩾18 years old, (2) whose clinical manifestations and pathologic biopsies were consistent with patients diagnosed with breast cancer, (3) with clear consciousness and could complete the questionnaire, and (4) who provided consent to participate in this study. Participants were excluded if they had been diagnosed with another illness or with psychiatric diseases.

Participants were asked to complete the sociodemographic questionnaire, the Chinese version of the Social Impact Scale, and the stigma items individually. The process lasted 15–25 min. A total of 50 patients completed the same items again, 2 weeks after the first test, to assess test–retest reliability. The sample size was at least 100 to ensure stability of the variance–covariance matrix (Terwee et al., 2007). Furthermore, the exploratory factor analysis requires a sample of 200 (Horne et al., 2017). Hence, we had to recruit more than 200 participants.

### Measures

Questionnaires with basic demographic information included age, gender, ethnicity, religion, place of residence, marital status, occupation, employment status, education level, family income status, medicare coverage, stage of cancer, and surgery type.

The 24-item Breast Cancer Scale contained 24 items. Participants rated their experiences of stigma by using a 4-point Likert-type scale (1 = “strongly disagree,” 4 = “strongly agree”). Scores ranged from 24 to 96. A higher score indicated a more significant stigma.

The Social Impact Scale (SIS) is a 24-item instrument (Fife and Wright, 2000) used to assess people’s stigma with HIV/AIDS and cancer. The responses to each item were based on a 4-point scale (“strongly disagree” to “strongly agree”). The score obtained on this scale was used as a criterion for the Breast Cancer Stigma Scale validity. The Chinese SIS is a widely used scale with good reliability (Pan et al., 2007). The separation reliability was 0.99, representing good internal consistency (Pan et al., 2007).

### Statistical Analyses

All statistical analyses were performed using the IBM SPSS 26.0 software, with an α of 0.05 for significance. General and disease-related characteristics were presented as numbers, mean, and *SD*. The validity evaluation of the scale included content, criterion, and construct validity, while the reliability evaluation comprised internal consistency and test–retest reliability.

### Validity

#### Content Validity

The item-level content validity index (I–CVI) was calculated for each item by dividing the number of experts who rated the item as reasonably necessary or highly important (i.e., a rating of 4 and 5 given by experts) by the total number of experts taking part in the rating (Polit et al., 2007). Cs represented experts’ familiarity with the research field, and Ca represented the judging criteria based on the experts. CV was defined as the SD divided by the mean, which is used to describe the relative dispersion degree of the item’s importance evaluation from experts (Reed et al., 2002; Chen et al., 2020). The Kendall coefficient *W*- test evaluated the consensus on agreement among the experts. It refers to the level of intra-expert understanding of all of the indicators (Xing et al., 2019). A two-tailed *p*-value of less than 0.05 was considered statistically significant (Chen et al., 2020). Therefore, we predefined a mean score of no less than 4, a CV of no more than 0.2, and a two-tailed *p*-value of Kendall coefficient *W*-test of no more than 0.05 among experts for the items to be included.

#### Discriminant Validity

Discriminant validity refers to a scale’s ability to distinguish between two or more groups (Li et al., 2019). Every participant received a total score after completing the questionnaire. Participants’ total scores on each item were listed in a sequence of numeric values from the lowest to highest for the assessment of internal criterion validity. The difference between the lower 27% and the upper 27% of the distribution was analyzed by an independent two-sample *t*-test. We deleted items that had a two-tailed *p*-value of ≥ 0.05 or *t* < 3.

#### Criterion Validity

The relationships between the Breast Cancer Stigma Scale scores and the Chinese version of the Social Impact Scale were examined to assess criterion validity. Pearson’s correlation coefficients (ρ) were considered no less than 0.3 (Salter et al., 2004).

#### Substantive Validity

Qualitative interviews, a literature review, and a theoretical model were used to explore subdimensions.

#### Construct Validity

Exploratory factor analysis (EFA) was used to assess the construct validity. The Kaiser–Meyer–Olkin index and Bartlett’s sphericity test were performed to determine the data suitability for EFA. Components were selected if eigenvalues were greater than 1. Items were considered for removal when their loading was less than 0.4 (Fu et al., 2015).

### Reliability

#### Internal Consistency

Internal consistency was assessed using Cronbach’s α. A Cronbach’s α of 0.7 or above was considered good internal reliability (Hendriks et al., 2013).

#### Split-Half Reliability

The split-half reliability of the scale was tested using the odd–even method. A correlation coefficient of 0.7 or above was considered good internal reliability.

#### Test–Retest Reliability

Test–retest reliability was examined through intra-class correlation coefficients (ICCs), represented by calculating Pearson’s ρ of two assessments for the same participant with an interval of 2 weeks in between assessments. An ICC greater than 0.70 suggests that the two tests had excellent test–retest reliability.

## Results

### Qualitative Interviews

Our sample consisted of 14 patients with breast cancer who sought treatment at the Hunan Cancer Hospital from July to September 2020. The characteristics of the participants are shown in Table 1. The authors analyzed the transcripts independently by bracketing data on preconceived ideas and strictly following the adapted Colaizzi’s method. Findings were then compared and discussed by the team until consensus on themes, theme clusters, and categories were achieved.

**TABLE 1:** Participant characteristics.

ID	Age	Place of residence	Marital status	Occupation	Education level	Payment	Stage of breast cancer	Approach of surgery
N1	48	Village	Married	–	Secondary school	Medical insurance	I	Modified radical mastectomy
N2	38	Village	Married	Worker	Primary school	New rural cooperative medical insurance	III	Modified radical mastectomy
N3	41	Township	Married	–	Secondary school	New rural cooperative medical insurance	II	Extensive radical surgery
N4	42	City	Married	Self-employed	University	New rural cooperative medical insurance	II	Breast reconstruction
N5	55	City	Married	Engineer	Technical secondary school	Medical insurance	III	Modified radical mastectomy
N6	41	City	Married	Worker	High school	Medical insurance	I	Modified radical mastectomy
N7	53	Township	Single	Worker	High school	New rural cooperative medical insurance	IV	Modified radical mastectomy
N8	47	Village	Married	Farmer	Primary school	New rural cooperative medical insurance	I	Modified radical mastectomy
N9	49	Village	Married	–	Primary school	New rural cooperative medical insurance	II	Modified radical mastectomy
N10	37	Village	Married	–	Secondary school	New rural cooperative medical insurance	IV	Modified radical mastectomy
N11	46	Village	Married	Civil servants	University	Medical insurance	III	Breast reconstruction
N12	41	Village	Married	Farmer	Secondary school	New rural cooperative medical insurance	I	Breast conservative operation
N13	36	Village	Married	Worker	Secondary school	New rural cooperative medical insurance	II	Modified radical mastectomy
N14	38	Township	Married	Housewife	Secondary school	Medical insurance	I	Modified radical mastectomy

#### Theme 1: Self-Image Disorder

Interview responses revealed that the body image of patients with breast cancer is damaged due to symptoms such as mass ulceration and breast asymmetry, and feeling of attraction decreases:

• Weight change—N1: “I used to be so thin. After taking hormone drugs, I gained 10 kg.”
• Hair loss—N1: “[after shaving her hair following chemotherapy since it resulted in alopecia] I bought a wig and brought it up; I am annoyed due to hair loss.”
  N2: “I always wear a wig at home.”
  N4: “I want to buy a beautiful wig.”
  N6: “I must wear a wig when I go out.”
  N10: “I care about hair loss since I am still young. I am a little troubled when I wear a wig.”
  N13: “My hair was badly lost after the second chemotherapy, I cried loudly when I shaved my hair because it is unacceptable. [My] chest is not integrated after the operation. I didn’t dare to go out a few days ago. I felt that shaving my head looks ugly; I heard that the eyebrows will fall out, although they haven’t fallen out yet, so I hurried to buy some eyebrow pens.”
• Image impairment—N1: “I don’t look as beautiful as before after dressing. I always pay attention to my image.”
• N5: “I’m still afraid to see my wound right now. Maybe the breast on the surgical side is like a man.”
• N6: “The breast is cut and becomes ugly; I regret not getting breast reconstruction surgery. The breast is really beautiful after reconstruction. Although it is painful, it just lasts for three months; otherwise, this (breast) will be gone for a lifetime. I thought that [it was good enough] as long as I was alive at first, but later, I found it ugly. The breast has been cut. The artificial breast is not as good-looking as the one on the other side. Additionally, it is hard. I want to make my breast more good-looking after I recover. Last time, a person [who underwent] breast reconstruction surgery showed her breast to me. It’s rather beautiful without a big scar. She can also wear a swimsuit.” [envious expression]
  N8: “I feel a little uncomfortable after cutting the breast. Everyone has breasts, but not me.”
  N10: “A little concerned about the lack of breasts.” [awkward smile]
  N12: “A little bit? [excited] So big! My wound is so big.”
  N13: “I care about the lack of breasts.” [raises the tone]
• Wear artificial breast—N1: “I bought an artificial breast online.”
• N6: “I have to wear a bra with an artificial breast; otherwise, I still feel a little bit strange.”

#### Theme 2: Social Isolation

Responses further revealed that patients with breast cancer avoid social contact due to their illness:

• N1: “I used to dance and go shopping, but now, I seldom go out. Some friends do not know I had breast cancer and asked me why I do not go out with them. I said that I work in Guangdong.” [angry and impatient]
• N2: “I do not want to work anymore. My family also does not want me to go to work [after my illness]. I rarely go out, even if I am invited out to play.”
• N6: “I am a patient now, not a healthy person. I haven’t gone back to work, and I do not want to go back to work. I wear a mask when I go out and do not want others to see me. I do not want to talk to others.”
• N10: “I have less contact with my friends. I can’t go to work anymore. I have to take good care of myself.”

#### Theme 3: Discrimination

Patients with breast cancer face discrimination because of changes in their social and family roles:

• N1: “A friend immediately blocked my WeChat after knowing that I had breast cancer. Some people will say, ‘Why you wear that hat? You look like a 70-year-old woman”’.
• N2: “My neighbor is rather boring. He went to my workplace inquiring about my illness.”
• N8: “Why is no one gossiping? Of course some people talk. ‘She had cancer.”’
• N13: “As soon as others hear about that cancer, they feel a little queer.”
• N14: “My husband asked me to get reconstruction surgery.”

#### Theme 4: Self-Perception

Patients with breast cancer experience humiliation and shame because they belittle their value or think they cannot achieve their goals:

• Depression and fear—N1: “I wish the tumor [had been] benign. My breast was cut off. I have no fun to live.”
  N2: “It has been diagnosed for so long, but I still feel more or less uncomfortable.” [wry smile]
  N3: “[At the time of diagnosis] I did not know much about the disease at that time. I was in a relatively low mood. I thought I would not have lived for long. It’s like I was sentenced to death.”
  N4: “When I was diagnosed, I felt like my life was over; it was like I was sentenced to death. Then, I was very flustered.”
  N7: “I did not believe the diagnosis.”
  N8: “I must be in a bad mood [sad]. It must be sad to be diagnosed. In the beginning, I could not accept it. I felt very miserable.”
  N10: “I could not believe and accept the diagnosis at the beginning.”
  N11: “I was confused when I was diagnosed.”
  N12: “Then, I broke down. At that time [when I was diagnosed], my tears flowed out. After the doctor left, I ran to the toilet crying for a while.”
  N13: “I cannot believe it. I just wonder how this disease must be on me. It is like a dream. I am still a little sad to say.”
  N14: “Diagnosis is a little unacceptable at first.”
• Conceal illness—N2: “My neighbor know [about] my disease. Many people do not know [about] my disease yet, and I do not want others know.”
  N4: “Because there were a lot of people [who] did not want to let others know [about] their disease.”
  N10: “Only family members and relatives knew [about the disease]; others did not.”
  N12: “I do not tell others. Only my friends and relatives know; others did not know. Anyway, I cannot let them know and do not want them to see me.”
  N13: “Few people know. My relatives know it. I cannot accept the disease, so I do not want to talk.”
• Hope to be a healthy person—N4: “I do not want others consider me as a patient. My breast was cut off; I do not want others to look at my breast intentionally or unintentionally to see what my breast looks like after it was cut off. Notably, it will still be a little uncomfortable and a little embarrassed. Only my relatives, the closest relatives, and immediate relatives know. Others do not know, and I do not want others to visit me or care about me. To have family members accompanying me is enough. It is meaningless, and I have to deal with them.”
• Worry about recurrence—N2: “Fear of recurrence; there is more or less a feeling of fear of recurrence.”
  N14: “Fear of proliferation.”
• Burden—N3: “I am still a little worried about the economic burden because it is long-term. My husband is busy. Additionally, if the treatment takes one year, I do not have much time to take care of the children and the elderly psychologically and physically. I’m a little worried.”
  N4: “My child is still young. If something happen to me, I will be a little stressed.”
  N6: “I feel that I am no longer as capable as before and have become a patient. Now, I’m sick. They have to take care of me and help me take care of my children.”
  N8: “I’m afraid of getting my family in trouble. I cannot do anything now. My family members have to take care of me.”
  N10: “It is inconvenient for me to move. I need someone else to take care of me.” [embarrassed] “I hope I can move by myself. My mother-in-law takes care of many things, which is very troublesome for my family members. I hope to recover as soon as possible without bothering them so that I can feel better. I spend less time accompanying and mentoring my child. I also feel sorry for my child.”
  N12: “Worry about the cost! I am afraid I cannot afford it; I feel remorseful because I have this disease.”
  N14: “The family is still a little burdened financially.”

### Delphi Study

Cs and Ca were, calculated to be 0.793 and 0.939, respectively. The mean value of the expert authority coefficient (Cr) was 0.866. The Kendall’s coefficient of concordance (W) was calculated to be 0.133–0.452 (*p* < 0.05). After two rounds of consultations, nine items were deleted, 19 were modified, and seven were merged. The initial 24-item scale was developed. I–CVI was calculated to be 0.73–1.0, and S–CVI/Ave was estimated to be 0.92.

### Sample Characteristics

A total of 218 questionnaires were distributed, and 200 valid questionnaires were recovered. The valid recovery rate was 91.74%. Respondents were between 29 and 62 years of age. Furthermore, the mean age was 45.405 years (*SD* = 6.55). Details are shown in Table 2.

**TABLE 2:** Sample characteristics.

Variable	Group	n	(%)
Age	18–44	99	49.5%
	45–54	86	43.0%
	≥55	15	7.5
Ethnicity	Ethnic Han	198	99.0
	Minority	2	1.0
Religion	Yes	2	1.0
	No	198	99.0
Place of residence	City	46	23.0
	Township	71	35.5
	Village	83	41.5
Marital status	Married	192	96.0
	Single	8	4.0
Occupation	Farmer	62	31.0
	Worker	36	18.0
	Civil servants	7	3.5
	Teachers	13	6.5
	Freelance professional	34	17.0
	Others	48	24.0
Employment status	Unemployed	86	43.0
	Employed	79	39.5
	Retired	13	6.5
	Others	22	11.0
Education level	Secondary school or below	125	62.5
	High school or technical secondary school	26	13.0
	College	27	13.5
	University or above	22	11
Family income status (RMB/month/per person)	≤1,000	48	24.0
	1,000–2,000	59	29.5
	2,000–5,000	80	40.0
	≥5,000	13	6.5
Medical insurance	Urban basic health insurance	1	0.5
	New rural cooperatives medical service	26	13.0
	Self-paying	113	56.5
	Others	60	30.0
Family history	Yes	13	6.5
	No	187	93.5
Stage of breast cancer	I	48	24.0
	II	95	47.5
	III	50	25.0
	IV	7	3.5
Type of surgery	Standard radical surgery	16	8.0
	modified radical mastectomy	112	56.0
	Extended radical surgery	19	9.5
	Mastectomy+breast reconstruction	43	21.5
	breast-conserving surgery	10	5.0
Adjuvant therapy	Chemotherapy	79	39.5
	Chemotherapy+Radiation therapy	15	7.5
	Chemotherapy+Hormone therapy	17	8.5
	Chemotherapy+ Targeted therapy	25	12.5
	Radiation therapy+Hormone therapy	1	0.5
	Hormone therapy	2	1.0
	Comprehensive therapy	49	24.5
	None	12	6.0
Time since diagnosis (months)	<1	1	0.5
	1–11	24	12
	12∼23	23	11.5
	24–35	139	69.5
	≥36	13	6.5
Time since surgery (months)	0∼	4	2
	1∼	30	15
	12’	19	9.5
	24∼	142	71
	36∼	5	2.5
Psychological counseling	Yes	5	2.5
	No	195	97.5

Two items (“My social activities have decreased because of my illness”[*t* = -2.881, *p* = 0.005] and “I felt miserable and emotionally devastated when diagnosed”[*t* = -1.373; *p* = 0.173]) were eliminated as they did not meet the criteria of discriminative validity. The participants’ total scores for each item were listed sequentially by numeric value (from the lowest to highest) for the assessment of internal criterion validity. The difference between the total scores of the lower 27% and the upper 27% of the distribution was analyzed by an independent two-sample *t*-test. We deleted items with a two-tailed *p* ≥ 0.05 or *t* < 3. Three items (“I feel bothered by chemotherapy-induced hair loss, pigmentation, and weight changes [*r* = 0.271, *p* < 0.01],” “My social activities have decreased because of my illness [*r* = 0.284, *p* < 0.01],” and “I felt miserable and emotionally devastated when diagnosed [*r* = 0.108, *p* < 0.01]”) were eliminated since there was a higher α on both the total scale and subscales. All *p* of inter-scale and inter-subscale correlation coefficients were < 0.01. The relationships between the scores of the Breast Cancer Stigma Scale and the Chinese version of the Social Impact Scale were examined to assess criterion validity. Pearson’s correlation coefficients were considered no less than 0.3. Therefore, we deleted items that were less than 0.3. Four items (“If I do not wear prosthetic breasts or take other measures, body asymmetry caused by surgery will make my center of gravity unstable,” “I feel bothered by chemotherapy-induced hair loss, pigmentation, and weight changes,” “My social activities have decreased because of my illness,” and “I felt miserable and emotionally devastated when diagnosed”) were eliminated. The Cronbach’s α coefficient method aims to observe the change in the reliability coefficient of the total quantity table after deleting an item. If the Cronbach’s coefficient of the total quantity table increases significantly after deleting an item compared with the original coefficient, it indicates that the item has low homogeneity with other items, and is deleted. In this study, the Cronbach’s coefficient of the total amount table was calculated first and then calculated after deleting an item. If the latter was greater than the former, the item was deleted. Hence, four items were deleted, resulting in a final scale of 20 items.

### Structural Validity

To identify the underlying components of the Breast Cancer Stigma Scale items, we performed two rounds of EFA. The data were suitable for EFA with a Kaiser–Meyer–Olkin (KMO) measure of sampling adequacy with a value of 0.789 and a highly statistically significant Bartlett Test of Sphericity (*P* < 0.0001). EFA revealed six eigenvalues greater than 1, explaining 67.50% of the variance. A Scree plot was used to examine changes in the eigenvalue. We explored changes in the eigenvalues by using the scree plot to determine the number of factors to be retained. A sharp drop was shown in the plotline slope after four factors. In addition, we considered the clinical significance, and deemed it inappropriate to exclude these items as they were all significant for the construct. Two eigenvalues and their items were deleted due to lesser theoretical correlation, and the items with loading below 0.40 (“My life and work were affected after my illness,” “If one looked down on me knowing that I was sick, I would hide him,” “I think the treatment makes my body incomplete,” “I feel self-blame because of the economic pressure and care pressure caused by my illness,” and “I was unable to take care of my family due to my illness”). The remaining 15 items were retained for further EFA. The KMO measure of sampling adequacy was 0.792 and was highly statistically significant. Bartlett Test of Sphericity suggested that the data were still suitable for EFA. Four factors were retained according to the inspection of the scree plot and contributed 69.443% to the explained variance. Details of the results of EFA are shown in Table 3.

**TABLE 3:** Item factor loadings (*n* = 200).

Items	Factor 1 Body-image impairment	Factor 2 Social isolation	Factor 3 Discrimination	Factor 4 Internalized stigma
1. I care about the changes to my breasts.	0.781			
2. I feel I am imperfect after surgery.	0.857			
3. I do not want to see or touch the scars left by surgery.	0.743			
4. After the surgery, I feel more anxious and less confident about my appearance than before.	0.849			
5. I feel the treatment has made me less physically attractive and less feminine.	0.846			
6. I do not think I am a healthy person.	0.428			
7. I cover my breasts when I am intimate with my partner.		0.767		
8. I am afraid of intimate physical contact, such as hugging.		0.845		
9. People usually sympathize with me because of my illness.			0.717	
10. I often feel people staring at me after my diagnosis.			0.951	
11. After my illness, I often hear people secretly talking about me after my diagnosis.			0.937	
12. I was ridiculed for wearing a hat due to the loss of hair caused by chemotherapy.			0.477	
13. I do not want anyone other than those closest to me to know I have been diagnosed with breast cancer.				0.834
14. I feel unnatural when someone looks at my chest.				0.729
15. I do not want anyone to see how I look after my illness.				0.841

### Reliability Assessment: Internal Consistency, Split-Half Reliability, and Test–Retest Reliability

As shown in Table 4, the Cronbach’s α coefficient, the split-half reliability coefficient, and the test-retest reliability coefficient for the 15-item Breast Cancer Stigma Scale and that of factors were all above 0.75. Table 4 elaborates on the reliability correlations of the Breast Cancer Stigma Scale.

**TABLE 4:** Reliability correlations for the Breast Cancer Stigma Scale (*N* = 200).

	Cronbach’s α	Split-half reliability	Test–retest reliability
Body-image impairment	0.882	0.903	0.919*
Social isolation	0.849	0.855	0.904*
Discrimination	0.750	0.803	0.884*
Internalized stigma	0.785	0.767	0.941*
Total	0.860	0.911	0.947*

*P < 0.01 (2-tailed).

### External Validity

Concerning external validity, we examined criterion validity. The correlation coefficient between the Breast Cancer Stigma Scale’s 15-item total score and the Chinese version of SIS’s 24-item average score was 0.641 (*p* < 0.001). There were significant correlations between the Breast Cancer Stigma Scale’s 15-items and all of the Chinese versions of SIS domains.

## Discussion

This study aimed to develop and verify the construct validity of the newly created the Breast Cancer Stigma Scale. The 15-item scale comprises four factors: self-image impairment, social isolation, discrimination, and internalized stigma. The scores of the expert consultation indicated that the scale has adequate content validity. Factor analysis findings suggest that the scale has an acceptable component construct. Other findings indicated highly estimated internal consistency, split-half reliability, and test–retest reliability. These results indicate that the 15-item Breast Cancer Stigma Scale is a valid and reliable instrument to assess stigma status in patients with breast cancer. Therefore, this Breast Cancer Stigma Scale can serve as a unique instrument for the assessment of perceived stigma among patients with breast cancer in China and potentially abroad.

Interviews revealed that stigma might emerge at different stages of a patient’s illness. Stigma is a significant contributor to low self-esteem, depression, and a tendency toward social isolation, which may hinder recovery at any stage of the illness, resulting in changes in social roles, acceptance, and challenges related to employment. Therefore, an accurate assessment of the stigma associated with breast cancer is in the patient’s best interest.

There is a strong linear correlation between the Breast Cancer Stigma Scale and the Social Impact Scale. The Social Impact Scale is a broad scale used for patients with all types of chronic illness, while the Breast Cancer Stigma Scale is used specifically for patients with breast cancer and is more unique in evaluating breast cancer-related stigma. Factor analysis indicated that a 15-item scale with four factors is optimal. Of the four factors of the Breast Cancer Stigma Scale, factors 1 (self-image impairment) and 4 (internalized stigma) were used to evaluate the self-stigma of patients with breast cancer. When coping with breast cancer, perceived stigma was assessed by factors 2 (social isolation) and 3 (discrimination). These findings were consistent with the definition of the stigma that we expounded in the Introduction. Furthermore, compared to 24 items on the Social Impact Scale, there are only 15 items on the Breast Cancer Stigma Scale. The shorter length of the new scale may improve completion rates with acceptable reliability and validity.

To the best of our knowledge, only one scale has been developed recently to assess stigma in patients with breast cancer. The Breast Cancer Stigma Scale for use with Arab patient populations (BCSS-A), consisting of a 12-item questionnaire, was using a sample of 59 women (Dewan et al., 2020). The Cronbach’s alpha coefficient of the BCSS-A was 0.79, the content validity of S-CVI was 1.0, and the item-CVI ranged from 0.85 to 1.0. The BCSS-A predominantly focuses on health-related stigma: perceived danger, blame, concealability, disruptiveness, esthetics, and shaming and devaluation of patients or their families. However, the total number of participants was 59, most of whom were married and on hormonal therapy. Therefore, it is difficult to generalize their findings to patients undergoing other treatments or single women. In this study, the total variance explained was 69.443%, and it was higher than the total variance of other cancer-related stigma scales.

Our study has several strengths. First, a significant strength of this research was that the scale was developed based on Chinese patients’ cultural context and experiences with breast cancer. Second, to ensure the integrity of the information and the scale, patients’ stigma status, conceptualization, and development of an initial item pool were based on various methods. Through a rigorous instrument development process and iterative scale validation, a reliable instrument has been tested for patients with breast cancer with a potentially stigmatized condition. Third, stigma was directly associated with patients’ mental health. Shame and embarrassment stemming from stigmatization may compromise patients’ body image and lead to psychological distress. The Breast Cancer Stigma Scale may provide a useful screening measure for identifying patients with a potentially stigmatized condition and provide those affected patients with appropriate psychological support.

There are some limitations to this study. First, we did not examine the confirmatory factor analysis of the Breast Cancer Stigma Scale. Therefore, it is important to confirm the factor structure in future studies. Second, the Breast Cancer Stigma Scale was developed based on the theory of perceived stigma. Focusing on perceived stigma only in patients with breast cancer was considered one of the strengths of the Breast Cancer Stigma Scale compared with the Social Impact Scale. However, more studies with larger sample sizes are needed to confirm this advantage. In addition, our study recruited participants conveniently and only from one hospital.

## Conclusion

The newly developed Breast Cancer Stigma Scale offers a valid and reliable instrument for assessing the stigma of patients with breast cancer in clinical and research settings. To the best of our knowledge, there has been no specific measurement of breast cancer-related stigma in China. The scale was tested and modified after a literature review, two rounds of Delphi panels, and qualitative interviews, thus capturing the spectrum of stigma relevant to patients with breast cancer. This study is a step forward for breast cancer stigma-related studies and provides a reference for developing effective interventions for those with potentially stigmatized conditions.

## Data Availability Statement

The raw data supporting the conclusions of this article will be made available from the corresponding author on reasonable request.

## Ethics Statement

The studies involving human participants were reviewed and approved by the Hunan Cancer Hospital Ethics Committee. The patients/participants provided their written informed consent to participate in this study.

## Author Contributions

XB, SL, AC, PN, XX, YX, and XL wrote the first draft of the manuscript. XB, SL, and XL involved in the data collection and statistical analysis. All authors reviewed the manuscript, contributed to critical changes, and approved the final version of the manuscript.

## Conflict of Interest

The authors declare that the research was conducted in the absence of any commercial or financial relationships that could be construed as a potential conflict of interest.

## Publisher’s Note

All claims expressed in this article are solely those of the authors and do not necessarily represent those of their affiliated organizations, or those of the publisher, the editors and the reviewers. Any product that may be evaluated in this article, or claim that may be made by its manufacturer, is not guaranteed or endorsed by the publisher.
