# Supplementary material for: Breast Cancer Stigma Scale: A Reliable and Valid Stigma Measure for Patients With Breast Cancer
Source: Front Psychol. 2022 Jun 10;13:841280. doi: 10.3389/fpsyg.2022.841280 (PMC9226439; doi:10.3389/fpsyg.2022.841280)
Supplement: Supplementary file 1 [file Presentation_1.pdf]

Appendix A.

Breast Cancer Stigma Scale

Items	Totally agree	Agree	Disagree	Totally disagree
**Self-image impairment**				
1.I care about the changes of my breasts.				
2.I feel I am imperfect after surgery.				
3.I do not want to see or touch the scars left by surgery.				
4.After the surgery, I feel more anxious and less confident about my appearance than before.				
5.I feel the treatment has made me less physically attractive and less feminine.				
6.I do not think I am a healthy person.				
**Social isolation**				
7.I cover my breasts when I am intimate with my partner.				
8.I am afraid of intimate physical contact, such as hugging.				
**Discrimination**				
9.People usually sympathize with me because of my illness.				
10.I often feel people staring at me after my diagnosis.				
11. After my illness, I often hear people secretly talking about me after my diagnosis.				
12.I was ridiculed for wearing a hat due to the loss of hair caused by chemotherapy.				
**Internalized stigma**				
13.I do not want anyone other than those closest to me to know I have been diagnosed with breast cancer.				
14.I feel unnatural when someone looks at my chest.				
15.I do not want anyone to see how I look after my illness.				

Appendix B.

乳腺癌患者病耻感量表

填表说明：以下是对乳腺癌患者特异病耻感量表的具体表述，表格中共有 15 句话，是想了解您对患病的真实想法，请您仔细阅读，根据是否同意每句话的说法，分别在“非常同意”、“同意”、“不同意”、“非常不同意”下划“√”。

内容	非常同意	同意	不同意	非常不同意
**自我形象紊乱**				
1.我在意自己乳房的变化				
2.我觉得乳房做了手术，自身就不完美了				
3.手术后，我不愿意看或触摸乳房手术留下的伤疤				
4.手术后，我比（手术）以前对自己的外在形象更焦虑无自信				
5.我觉得治疗让我外表吸引力减少了，不再那么女性化了				
6.我觉得自己不是一个健康人				
**社会隔离**				
7.在与伴侣亲密时，我会遮住胸部				
8.我害怕亲密的身体接触：如拥抱等				
**歧视**				
9.因为患病，人们通常会同情我				
10.患病后，我常常感觉有人盯着我看				
11.患病后，我常常听到有人背后偷偷议论我				
12.因为化疗所致脱发而带帽子后被嘲笑				
**自我感受**				
13.我不想让除了亲近之外的人知道我患有乳腺癌				
14.当别人看我胸部时，我感觉不自然				
15.我不想让别人看到我患病后的样子				
